# A Preliminary Investigation on the Statistical Correlations between SARS-CoV-2 Spread and Local Meteorology

**DOI:** 10.3390/ijerph17114051

**Published:** 2020-06-05

**Authors:** Giorgio Passerini, Enrico Mancinelli, Mauro Morichetti, Simone Virgili, Umberto Rizza

**Affiliations:** 1 Department of Industrial Engineering and Mathematical Sciences, Marche Polytechnic University, 60131 Ancona, Italy; g.passerini@univpm.it (G.P.); s.virgili@staff.univpm.it (S.V.); 2 Institute of Atmospheric Sciences and Climate, National Research Council, 73100 Lecce, Italy; m.morichetti@isac.cnr.it (M.M.); u.rizza@isac.cnr.it (U.R.)

**Keywords:** COVID-19, COVID-19 incidence, SARS-CoV-2, meteorology, outdoor temperature, outdoor relative humidity

## Abstract

The statistical correlation between meteorological parameters and the spread of Coronavirus Disease-2019 (COVID-19) was investigated in five provinces of Italy selected according to the number of infected individuals and the different trends of infection in the early stages of the epidemic: Bergamo and Brescia showed some of the highest trends of infections while nearby Cremona and Mantova, showed lower trends. Pesaro–Urbino province was included for further investigation as it was comparably affected by the epidemic despite being the area far from the Po valley. Moving means of the variables were considered to take into account the variability of incubation periods and uncertainties in the epidemiological data. The same analyzes were performed normalizing the number of new daily cases based on the number of checks performed. For each province, the moving mean of adjusted and unadjusted new daily cases were independently plotted versus each meteorological parameter, and linear regressions were determined in the period from 29th of February 2020 to 29th of March 2020. Strong positive correlations were observed between new cases and temperatures within three provinces representing 86.5% of the contagions. Strong negative correlations were observed between the moving means of new cases and relative humidity values for four provinces and more than 90% of the contagions.

## 1. Introduction

On 31th of December 2019, pneumonia of unknown etiology was first reported by the offices of the World Health Organization (WHO) in China [1]. This disease was labelled as Coronavirus Disease-2019 (COVID-19) [2]. Based on phylogeny, taxonomy, and established practice, Gorbalenya et al. [3] have marked the related virus of COVID-19 disease as severe acute respiratory syndrome Coronavirus 2 (SARS-CoV-2). SARS-CoV-2, as in the case of another zoonotic coronavirus originated in the early 2000s with pandemic potential [4], was first transmitted from animals to humans [1].

At present, the human-to-human transmission of the COVID-19 virus has been reported via close unprotected contact with infected droplets and fomites, whereas the airborne transmission is considered possible only for specific circumstances and settings in the hospital context [5].

In the early 2000s, SARS transmission was controlled through quarantine, social distancing, travel restrictions, and contact precautions since no specific vaccines or drugs existed for this new pathogen to human beings [6]. Building on the experience of the Chinese government, the Italian government adopted control measures for the containment of the COVID-19 epidemiological emergency. In order to prevent the virus from extending beyond a few affected areas in Italy, various activities and daily life were regulated with a series of Decrees of the President of the Council of Ministers (DPCM) (mainly [7,8,9]). Since the COVID-19 outbreak became much more severe, the Italian government imposed a nationwide lockdown through another DPCM [10].

Since little is known about COVID-19 disease and its causative virus, the scientific community responded to the dramatic emergency posed by the COVID-19 pandemic shifting research focus to the COVID-19 related issues. Environmental constraints are expected to influence human coronavirus outbreaks, as environmental survival factors have been defined for other viruses in the past [11,12]. Therefore, a number of studies have examined the relation between the incidence of COVID-19 and atmospheric variables such as surface temperature, relative humidity, air pressure, ultraviolet radiation, wind speed, and atmospheric particles [13,14,15,16,17,18].

Considering air temperature and relative humidity, both the 2002 and 2019 SARS epidemics originated during cold and particularly dry winters in China [19]. Sajadi et al. [16] have observed that seven cities around the globe with latitudes in the range 30–50 N′′ and affected by the early onset of COVID-19 pandemic had similar average values of temperature (5–11 °C) and relative humidity (47–79%) both monitored at 2 m height.

Several authors [15,17,20], have found linear relationships between climatic variables (i.e., relative humidity and temperature) and COVID-19 incidence. Based on the analysis of more than 100 countries, Islam et al. [15] have observed that temperature and relative humidity were inversely associated with the incidence rate of COVID-19, thus suggesting that cold and dry climate favors virus survival. Wang et al. [17] analyzed the early onset of the COVID-19 outbreak in 100 Chinese cities considering mean values of temperature and relative humidity in the range from −21 to 21 °C and from 47% to 100%, respectively. These authors have found negative relationships between relative humidity, temperature, and the daily effective reproductive number for COVID-19, although with coefficient of determinations of about 20%. Rahman et al. [20] have confirmed a negative correlation between the positive cases of 12 Chinese provinces and median daily temperature, whereas no clear trend can be observed for the number of infected versus relative humidity.

Ficetola and Rubolini [14] reported nonlinear relationships between COVID-19 growth rates and climatic variables such as temperature and absolute humidity, with peaks in regions with mean temperature of about 5 °C and absolute humidity in the range of 0.6–1 kPa. A nonlinear relationship between temperature and SARS-CoV-2 transmission was reported also by Wang et al. [21].

According to Chen et al. [13], the optimal climate conditions for SARS-CoV2 transmission occur at a temperature of around 8 °C and relative humidity in the range 60–90%. A similar value of the average temperature (8.7 °C) was identified as optimal for SARS-CoV2 transmission also in the work of Wang et al. [21].

However, Yao et al. [18] have reported no significant associations between daily mean temperatures and the spread ability of SARS-CoV-2 in Chinese cities, thus concluding that temperature is not a driving factor of SARS-CoV-2 transmissibility. O’Reilly et al. [22] have observed that the countries with reported local SARS-CoV-2 transmission had a wide range of temperature and relative humidity, thus excluding seasonality as key modulating factor of SARS-CoV-2 transmissibility. Furthermore, there is a risk for misinterpreting the covariance between the incidence of infectious diseases and noncausal phenomena because of their similar seasonal pattern [23]. Other factors such as human population density may better explain the spatial distribution pattern of COVID-19 cases [24].

The aim of the present work is to investigate the statistical correlation between meteorological parameters (i.e., daily mean temperature, and relative humidity) and the incidence of COVID-19 in Italy.

The time period considered was from 29th of February 2020 to 29th of March 2020. In this time period, Italy was the hardest hit country around the globe with more than 90,000 reported positive cases [25]. The emotional impact of the situation posed by the COVID-19 emergency recall the line ‘’no man is an island entire of itself; every man is …, a part of the main’’ by John Donne [26]. Five provinces were selected according to the number of infected individuals in the early stages of the COVID-19 epidemic in Italy. Furthermore, these provinces represent three different climatic areas of Italy.

The fact that this study is limited to the analysis of meteorological parameters does not imply that these are considered as main driving factors of the COVID-19 incidence. It is likely that several factors, other than the meteorological ones, concur in determining the incidence of COVID-19.

## 2. Materials and Methods

**Figure 1:**
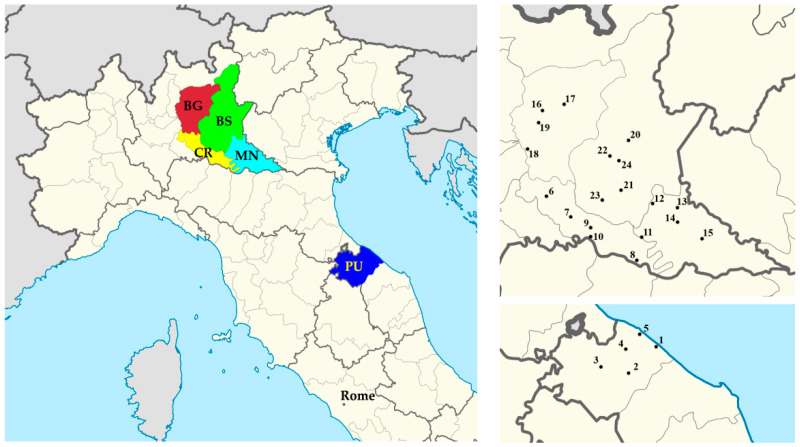
Left side: the five provinces selected for this study were the most affected by COVID-19 during March 2020. Bergamo (BG), Brescia (BS), Cremona (CR), and Mantova (MN) belong to Lombardy and are all within Po Valley while Pesaro–Urbino (PU) belongs to Marche and is located on the Mid-Adriatic coast. Right side: the cities selected to extract the simulated meteorological data in Lombardy and Marche. The cities are 1—Fano, 2—Fossombrone, 3—Urbino, 4—Vallefoglia, and 5—Pesaro, 6—Crema, 7—Soresina, 8—Casalmaggiore, 9—Castelverde, 10—Cremona, 11—Canneto sull’Oglio, 12—Castel Goffredo, 13—Volta Mantovana, 14—Goito, 15—Mantova, 16—Alzano Lombardo, 17—Cene, 18—Brembate, 19—Bergamo, 20—Lumezzane, 21—Ghedi, 22—Ospitaletto, 23—Manebrio, and 24—Brescia.

In the present study, we considered four provinces located in northwest Italy within the Lombardy region: Bergamo (BG) Brescia (BS), Cremona (CR), and Mantova or Mantua (MN) (Figure 1). We also considered one province located in central Italy within Marche region: Pesaro–Urbino (PU). The five provinces were selected according to the number of infected individuals in the early stages of the COVID-19 epidemic in Italy. In particular, BG and BS showed some of the highest trends of contagion, while CR and MN showed rather lower trends and were selected to check statistical evidence under different conditions (Table 1). Finally, Pesaro–Urbino province was included for further investigation as it was comparably affected by the epidemic despite being the area far from the Po valley. Here, the orography is more complex, and the province includes a mountainous area and a valley-coastal environment. However, most of the population lives near the coast, so for this study, we employed temperatures observed near the city of Pesaro, located on the coast.

### 2.1. Measured and Modelled Meteorological Time Series

The five provinces belong to three different climatic regions with slight differences in mean annual values of meteorological parameters [27]:

(i) Mediterranean suboceanic to subcontinental, influenced by mountains climate for BG;
(ii) Mediterranean suboceanic to Mediterranean subcontinental climate for CR, BS, and MN;
(iii) Mediterranean suboceanic climate for PU.

Daily average temperature and relative humidity were downloaded from the web sites of Regional Agency for the Environmental Protection of Lombardy [28] and the regional agrometeorological service of Marche [29]. In each province, a single monitoring site, located nearby the related capitals, was considered representative of the meteorology of the whole area (Table 2).

This was considered important to avoid weakening of experimental statistics. However, to confirm such representativeness, a meteorological simulation was carried through the Weather Research and Forecasting (WRF) model version 4.1.3 [30].

The WRF model was applied from 1 February 2020 (00:00 UTC) to 1 April 2020 (00:00 UTC), with a simulation domain covering the entire Italian peninsula having 240 × 270 grid points with a horizontal grid spacing of 10 km for both directions (west-east and south-north) and 40 vertical levels up to 50 hPa. The simulated daily mean temperature and relative humidity were evaluated in at least four cities for each province, collecting a total number of 24 points (Figure 1). The physical parameterization of the WRF model for this domain was set following Rizza et al. [31].

**Figure 2:**
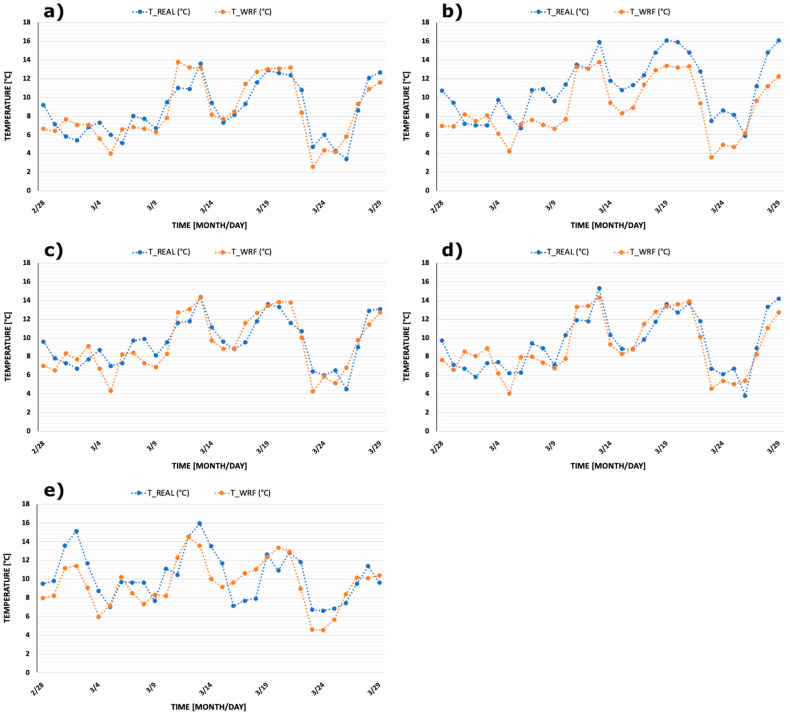
Comparisons between daily mean temperatures measured at selected province monitoring stations (‘T_REAL’) and the related average over each province obtained through the Weather Research and Forecasting (WRF) model (‘T_WRF’). Provinces are: (**a**) Bergamo, (**b**) Brescia, (**c**) Mantova, (**d**) Cremona, and (**e**) Pesaro Urbino.

**Figure 3:**
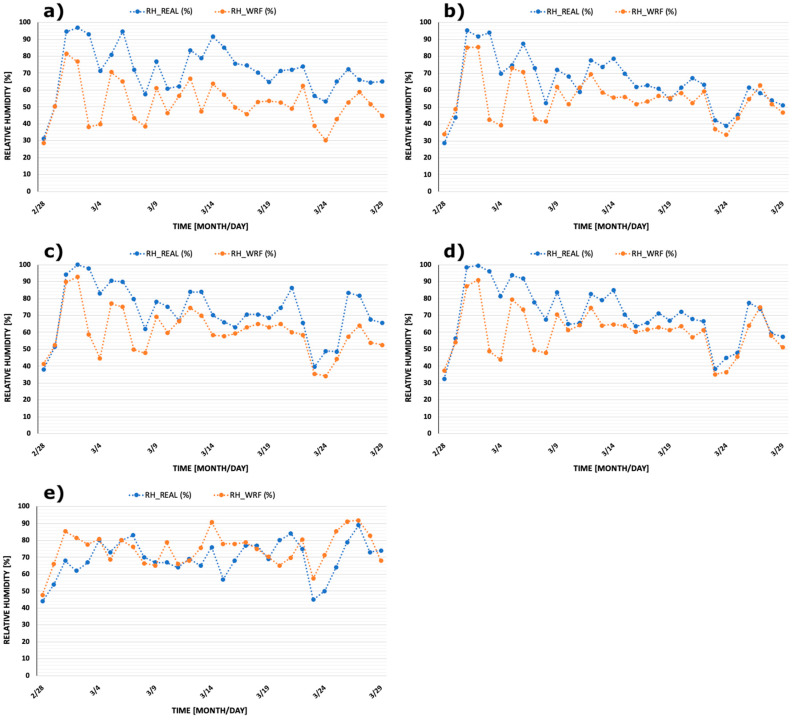
Comparisons between daily mean relative humidity measured at selected province monitoring stations (‘RH_REAL’) and the related average over each province obtained through the WRF model (‘RH_WRF’). Provinces are: (**a**) Bergamo, (**b**) Brescia, (**c**) Mantova, (**d**) Cremona, and (**e**) Pesaro Urbino.

For each province, the average of such values were calculated as arithmetic means, resulting in single daily values to be compared with the daily mean values measured at the monitoring stations of the respective capitals (Table 2). Figure 2 and Figure 3 show comparisons between measured and modelled daily mean temperature and relative humidity for the five provinces. Monitored data and model results show very similar trends although, especially for relative humidity, few discrepancies are readily evident. However, in almost all cases, the trends of the two sets of time series are very similar. Therefore, the selected monitoring stations can be considered representative for the meteorology of the whole area of each province for the time period considered.

### 2.2. Epidemiological Data

Epidemiological data were downloaded from the Italian Civil Protection web site [32]. Original data, published day by day, consisted of the overall amount of infected individuals for each province and the total number of individuals checked, and individuals infected for each region. From such data, we determined the number of new infected individuals within each province and the number of individuals checked and infected within each region for each day from the 29th of February 2020 to the 29th of March 2020. Moving means were calculated over 5 days and 8 days, since the statistics of the new daily positive cases may be affected by the length of the incubation period and discrepancies in the epidemiological data.

A five-day moving mean was adopted for all statistical variables as 5 days is the average incubation period for Covid-19 disease [1]. In fact, WHO [1] reported a mean incubation period of 5–6 days, and a range 1–14 days. However, according to Lauer et al. [33], 97.5% of the individuals that develop symptoms will do so within 11.5 days (confidence interval, 8.2–15.6 days) of infection. Thus, also an 8-day moving mean was adopted in order to take into account possible longer time required for symptoms to appear, possible delays between the arising of symptoms and testing for the disease, possible discrepancies in the epidemiological data from the COVID-19 outbreak because of variations in the daily number of specimen collected, possible diverse testing criteria, and the possible discrepancies from the date of specimen collection and the release of the test result.

As pointed out in previous studies regarding the onset of COVID-19 outbreak in Italy [34,35], the epidemiological data could be affected by errors both at regional and at national level. Previous studies [13,15] have considered a time lag of 7–14 days between the meteorological variables and the incidence of COVID-19. However, we considered the moving mean a better option for the analysis of data subject to the abovementioned uncertainties.

The number of cases was considered both as a raw variable and “adjusted” by the number of checks performed during the same period. To take into account the time needed to carry out the tests, the related values were computed shifting the time period one day back.

For example, the number of individuals infected between 10th and 15th of March was normalized by the number of checks performed between 9th and 14th of March. The use of the term “adjusted” instead of the more customary “normalized” or “biased” is due to the unfortunate circumstance that the number of tests performed daily is not available at province level but only for each Italian region. Thus, we opted for the following procedure in order to adjust the number of new daily positive cases based on the daily number of specimens collected at region level. By considering per each province the ratio between the new daily positive cases within the related region and the total number of tests performed within the same region, the adjusted new daily cases (*n*-day moving mean, with *n* = 5, or *n* = 8) was calculated for each province as follows:

(1) $$\mathrm{Adjusted}\;\mathrm{daily}\;\mathrm{cases}={\left(\sum_{i=1}^{n}\frac{{\left(\mathrm{new}\;\mathrm{daily}\;\mathrm{cases}\;\mathrm{in}\;\mathrm{the}\;\mathrm{province}\;\right)}_{i}}{n}\right)}_{j}\cdot \left(\sum_{i=1}^{n}\frac{1}{n}\frac{{\left(\mathrm{new}\;\mathrm{daily}\;\mathrm{cases}\;\mathrm{in}\;\mathrm{the}\;\mathrm{region}\;\right)}_{i}}{{\mathrm{number}\;\mathrm{of}\;\mathrm{tests}\;\mathrm{in}\;\mathrm{the}\;\mathrm{region}\;}_{i-1}}\;\right)$$

where *i* and *j* are indexes in the range 1–30, covering the time period 29th of February 2020 to 29th of March 2020; ${\mathrm{new}\;\mathrm{daily}\;\mathrm{cases}\;\mathrm{in}\;\mathrm{the}\;\mathrm{province}}_{i}$—number of new daily cases reported in each province on the *i*-th day; ${\mathrm{new}\;\mathrm{daily}\;\mathrm{cases}\;\mathrm{in}\;\mathrm{the}\;\mathrm{region}}_{i}$—number of new daily cases reported in the respective region on the *i*-th day; ${\mathrm{number}\;\mathrm{of}\;\mathrm{tests}\;\mathrm{in}\;\mathrm{the}\;\mathrm{region}}_{i-1}$ number of tests performed in each region on the (*i*−*1*)-th day. The indices (*i*−*1*) take into account the time shift postulated between the sampling phase and the communication of results.

### 2.3. Statistical Methods

The Pearson correlation coefficient (PCC) was calculated to evaluate possible relations between the moving means of the meteorological parameters (i.e., temperature, and relative humidity) and the moving mean of adjusted/unadjusted new daily cases. PCC is a number between −1 and 1 (following the Cauchy–Schwarz inequality) that determines the possible linear correlation between two variables. When PCC = 1, there is a complete positive linear correlation; when PCC = 0, there is no linear correlation; and when PCC = −1, there is complete negative linear correlation.

When checking the linear correlation between two data sets having the same number of samples (e.g., time series of experimental variables *X* and *Y*, randomly collected in the number of *n*), the PCC, often labelled as ‘R’, may be seen as:

(2) $$R=\frac{\mathrm{cov}\left(X,Y\right)}{\sigma_{X}\cdot \sigma_{Y}}$$

where cov(X,Y) is the covariance of $\left(X,Y\right)=1/n\sum_{i=1}^{n}\left[\left(X_{i}-\bar{X}\right)\times \left(Y_{i}-\bar{Y}\right)\right]$, $\bar{X}$ and $\bar{Y}$ being the ‘expected’ (mean) values of the two variables, while $\sigma_{X}$ and $\sigma_{Y}$ are the standard deviations as, for example, $\sigma_{X}={\left[\overset{n}{\underset{i=1}{\sum }}{\left(X_{i}-\bar{X}\right)}^{2}\right]}^{1/2}$.

In simple terms, PCC assumes positive values whenever, within two time series, high values of the first series mostly correspond to high values of the second series. On the other hand, PCC assumes negative values whenever, within two time series, high values of the first series mostly correspond to low values of the second series. Finally, PCC assumes values close to zero whenever no such conditions are verified. It is important to avoid confusing correlation with causation. When two variables are correlated, there may or may not be a causative connection, and this connection may moreover be indirect. Correlation can be interpreted in terms of causation only when the variables under investigation provide further logical, biological, or physical foundations for such interpretation.

To validate the statistical correlation of two variables or time series, the ‘*p*-value’ (probability value) is customarily employed. In a statistical investigation, the *p*-value can be seen as the probability of obtaining statistics at least as significant as the ones obtained, assuming that the so-called ‘null hypothesis’ is correct. The ‘null hypothesis’ is a statement that theorizes no statistical relationship between two observed phenomena and, thus, between the two related time series.

In simple terms, the null hypothesis implies that no linear relationship exists between variables, and the *p*-value can be seen as the probability that the current results would be found if the correlation were, in fact, null (which is exactly within the null hypothesis). Conventionally, if the probability value is lower than 5% (*p*-value < 0.05) the correlation coefficient may be called statistically significant [36].

For this study, Pearson’s analyses were performed over moving mean of meteorological parameters as the first time series *X* and the moving mean of “unadjusted” new daily cases or “adjusted” new daily cases as the second time series *Y*. Most of statistical analyses were carried out using the ‘R Project for Statistical Computing’ version 3.3.4 [37].

## 3. Results

We checked several meteorological parameters, namely outdoor temperature, relative humidity, wind speed and wind direction. However, statistical analysis of wind data was not significant, which was likely due to the narrow range of wind speed values (namely 0.5–2 ms^−1^) and the sparse distribution of wind direction data. Therefore, here we present the results of Pearson’s analysis related to outdoor temperature and relative humidity. In fact, only for these meteorological parameters, results showed significant statistical correlations, and in most cases related probability values were much lower than 5% [36]. Here, we present Pearson’s analyses performed over eight couples of experimental data series. The most inclusive and impressive results are the values of PCC found for the time series related to the number of new daily infected individuals within the five selected Italian provinces, the daily mean temperatures as monitored at the related capitals, and the daily mean relative humidity values observed at the same stations. As above outlined, we chose to use actual data monitored nearby province capitals, but we checked by means of a meteorological model the representativeness of such data within most populated areas of the entire related provinces. The time series are related to a 30-day time period from 29th of February 2020 to 29th of March 2020 and were averaged through 5-day moving mean and 8-day moving mean.

Figure 4, Figure 5, Figure 6, Figure 7, Figure 8, Figure 9, Figure 10 and Figure 11 show on the abscissa axis a meteorological variable and on the ordinate axis the number of new infections.

Each dot corresponds to the situation on a certain time period (5 days or 8 days) and the last day of such period is reported through the corresponding label. Thus, each dot represents a couple of values (X,Y) belonging to the two related time series. For instance, *X* could be the average number of new infections during five days while *Y* could be the mean daily temperature averaged over the same days. As already outlined, the number of cases was considered both as a raw variable and “adjusted” by the number of checks performed during the same period. Each figure also shows the regression line related to those two data sets, the value of PCC as ‘R’, and the related *p*-value as ‘*p*’.

### 3.1. Statistics of New Cases of COVID-19 vs. Outdoor Temperatures in Five Italian Provinces

**Figure 4:**
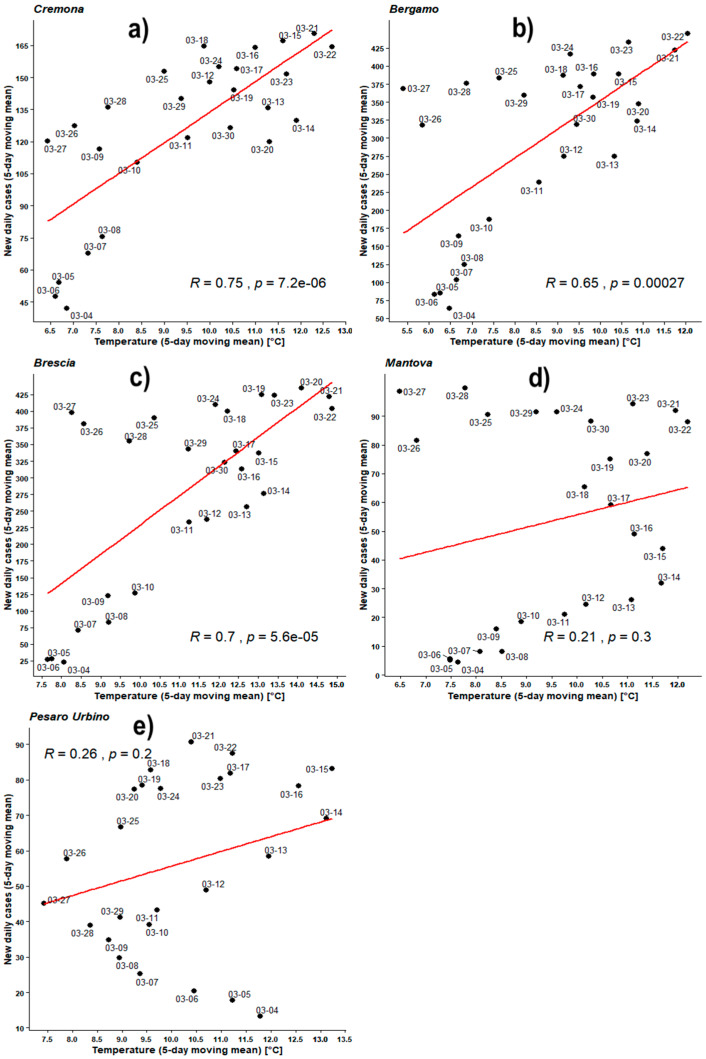
Scatter plot and linear regression for the 5-day moving means of new daily cases of COVID-19 against outdoor temperatures for five Italian provinces: (**a**) Cremona, (**b**) Bergamo, (**c**) Brescia, (**d**) Mantova, (**e**) Pesaro Urbino. Labels report, in MM-DD format, the last day of the moving means. Pearson correlation coefficient is reported as ‘R’ while probability value is reported as ‘p’.

Figure 4 shows the correlations between the number of recently infected people and the related outdoor mean temperatures. Temperatures range between 5 and 15 °C while mean total new daily infections are up to about 500/day in BG and BS, up to 150/day in CR and up to 100/day in MN and PU. All panels of Figure 4 show positive correlations, namely, the PCCs (R) of BG, BS, and CR in the range of 0.65–0.75 with *p*-values much lower than 0.05 corroborating such results.

On the other hand, MN and PU show much lower Pearson correlation values (namely 0.21 and 0.26, respectively) associated with *p*-values much higher than previous ones. This allows us to assume that the related results cannot be considered of statistical importance.

However, MN and PU registered lower amounts of infections and a different approach to patients’ checks, so we decided to adjust the daily number of individuals found infected in each province by the number of checks performed in the respective region.

**Figure 5:**
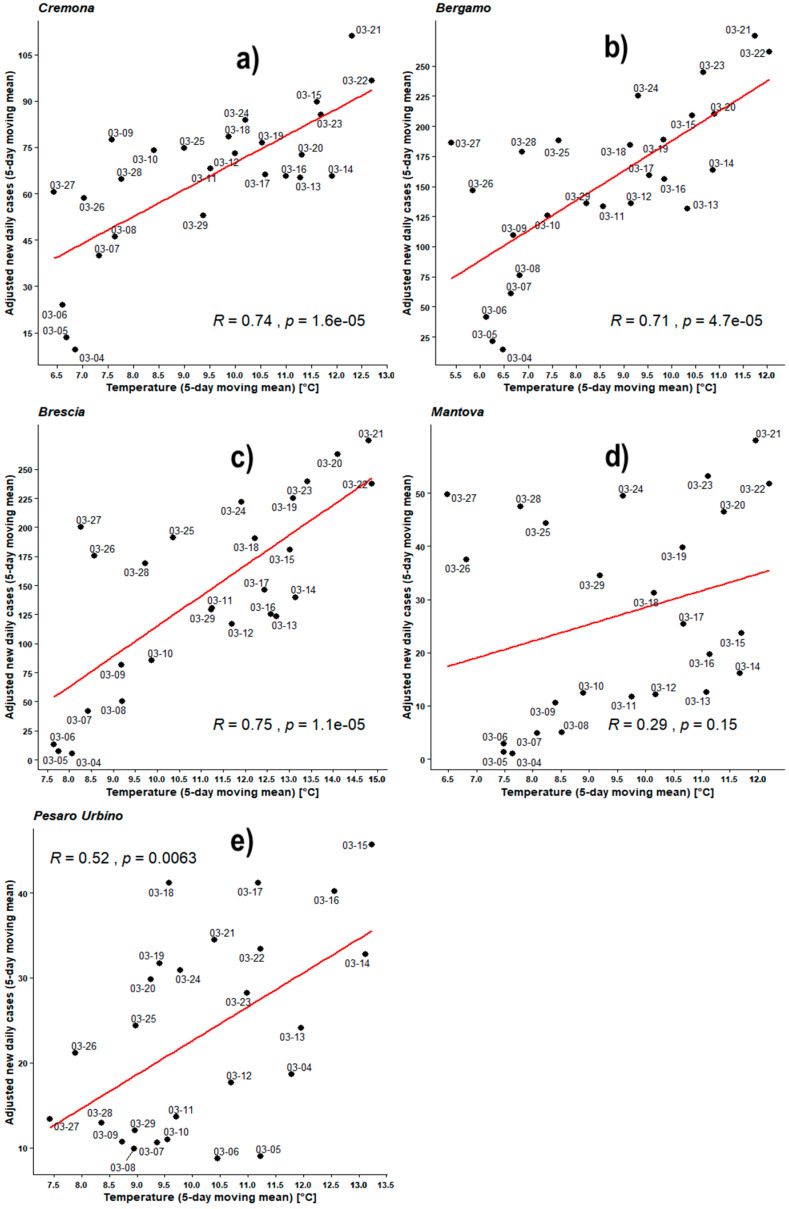
Scatter plot and linear regression for the 5-day moving means of adjusted new daily cases of COVID-19 against outdoor temperatures for five Italian provinces: (**a**) Cremona, (**b**) Bergamo, (**c**) Brescia, (**d**) Mantova, (**e**) Pesaro Urbino. Labels report, in MM-DD format, the last day of the moving means. Pearson correlation coefficient is reported as ‘R’ while probability value is reported as ‘*p*’.

Figure 5 shows Pearson’s analyses regarding the 5-day moving mean of daily new infected individuals, adjusted against the number of individuals checked in the corresponding regions, and the 5-day moving mean of outdoor daily mean temperatures. All PCC are positive and the values of BG, BS, and CR are very similar, in a range of 0.71–0.75 with *p*-values all much lower than 0.05 but also very similar, in the range 1.6–4.7 × 10^−5^. This time PU shows a PCC = 0.52 and a *p*-value now down to 0.0063.

Finally, MN’s statistics do not change at all, still showing a statistical behavior near to the so-called null hypothesis.

A first conclusion may be raised here. Both analyzing new cases and analyzing adjusted new cases, linear correlations exist and are always positive in the temperature range 5–14 °C, and statistically significant at least in three most infected provinces. When normalizing the number of infected people by the number of checks performed, results tend to equalize and become very similar, almost equal, for the three most infected provinces (BG, BS, and CR). PU is now much closer to the Padania’s statistics, but the significance of the related analysis is much lower with its *p*-value only one order of magnitude lower than the acceptable value. MN’s statistics are very close to the null hypothesis.

**Figure 6:**
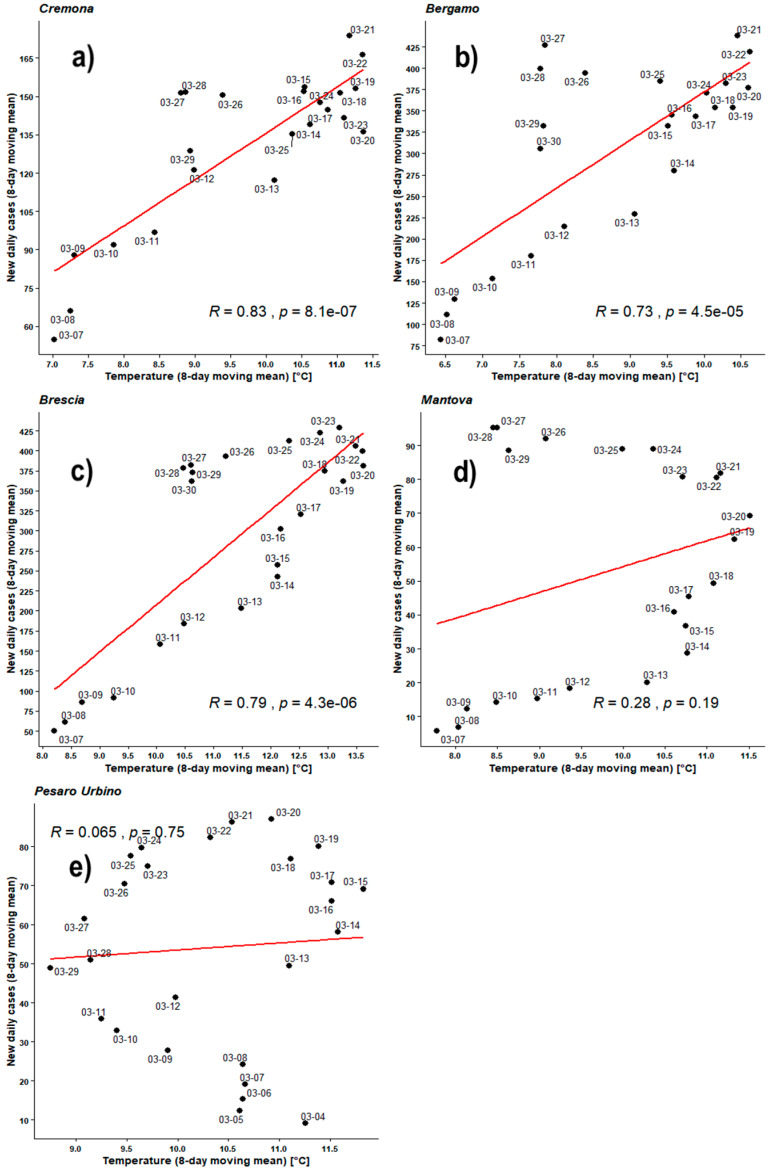
Scatter plot and linear regression for the 8-day moving means of new daily cases of COVID-19 against outdoor temperatures for five Italian provinces: (**a**) Cremona, (**b**) Bergamo, (**c**) Brescia, (**d**) Mantova, (**e**) Pesaro Urbino. Labels report, in MM-DD format, the last day of the moving means. Pearson correlation coefficient is reported as ‘R’ while probability value is reported as ‘*p*’.

Figure 6 shows the Pearson’s analyses employing the 8-day moving mean both for outdoor temperatures and for new infections. All PCC are positive in the temperature range 5–14 °C. As expected, all values remain very similar to those obtained applying the 5-day moving mean. For BG, BS, and CR PCCs are in the range 0.73–0.83 with very good *p*-values. MN and PU keep their behavior and remain very close to the null hypothesis.

**Figure 7:**
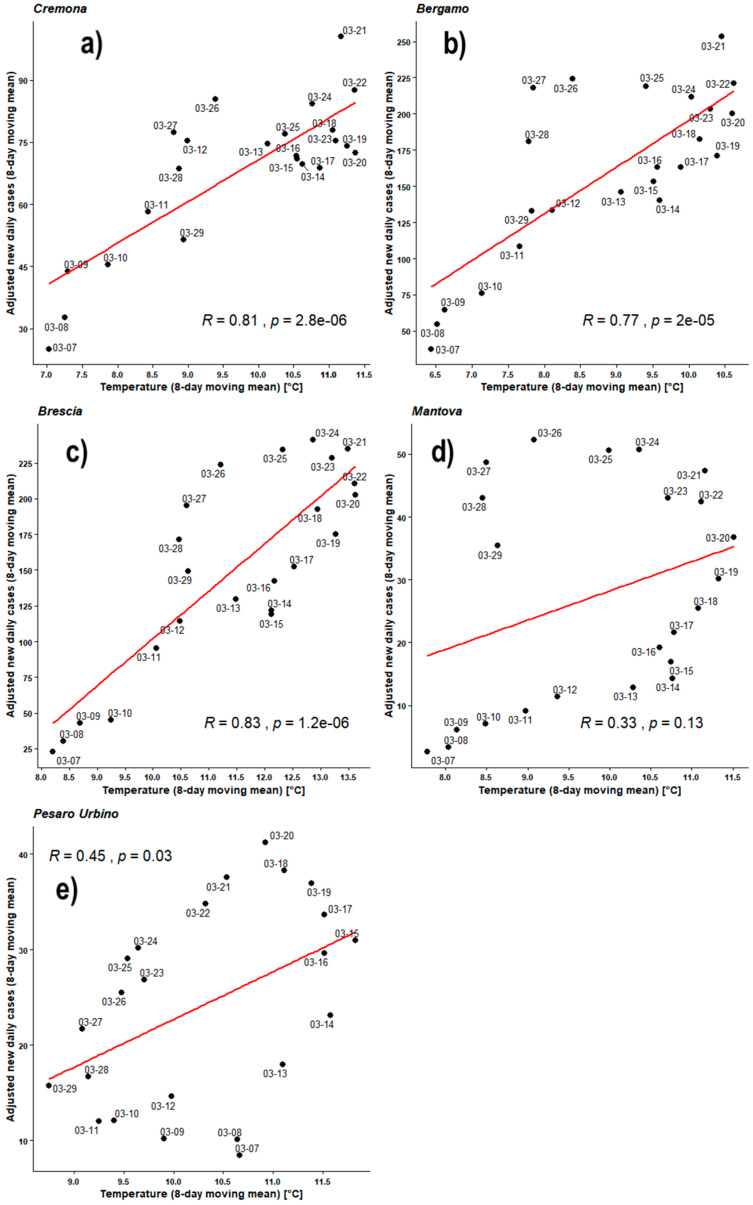
Scatter plot and linear regression for the 8-day moving means of adjusted new daily cases of COVID-19 against outdoor temperatures for five Italian provinces: (**a**) Cremona, (**b**) Bergamo, (**c**) Brescia, (**d**) Mantova, (**e**) Pesaro Urbino. Labels report, in MM-DD format, the last day of the moving means. Pearson correlation coefficient is reported as ‘R’ while probability value is reported as ‘*p*’.

Figure 7 shows the Pearson’s analyses employing the 8-day moving mean both for outdoor temperatures and for adjusted new infections. All PCC are positive in the temperature range 5–14°C. PCCs of BG, BS, and CR are in the range 0.77–0.83 with very good *p*-values. PU has now a fair PCC = 0.45 but the *p*-value = 0.03 is just below the significance level. MN keeps a low PCC = 0.33 associated with *p*-value much higher than the significance level, implying that the data distribution cannot be linearly correlated.

### 3.2. Statistics of New Cases of COVID-19 vs. Outdoor Relative Humidity in Five Italian Provinces

**Figure 8:**
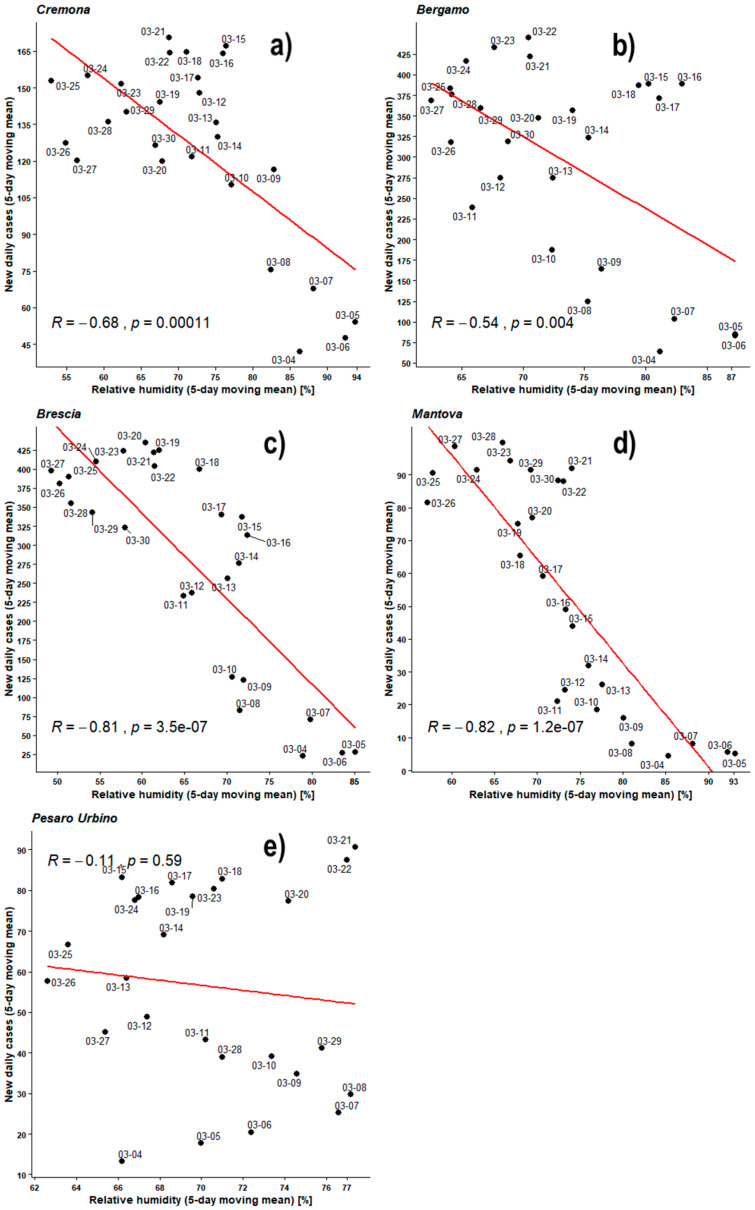
Scatter plot and linear regression for the 5-day moving means of new daily cases of COVID-19 against relative humidity for five Italian provinces: (**a**) Cremona, (**b**) Bergamo, (**c**) Brescia, (**d**) Mantova, (**e**) Pesaro Urbino. Labels report, in MM-DD format, the last day of the moving means. Pearson correlation coefficient is reported as ‘R’ while probability value is reported as ‘*p*’.

Figure 8 shows the Pearson’s analyses performed over daily average relative humidity time series and recently infected individual time series both averaged by the 5-day moving mean.

The mean amounts of infections obviously remain up to about 500/day in BG and BS, up to 150/day in CR and up to 100/day in MN and PU while average humidity ranges are 50–95%, but for PU that shows a narrower range about 65–75%.

All observed PCCs are negative in the relative humidity range 50–95%. The PCCs of BG, BS, CR, and MN are between −0.54 and −0.82 while all the *p*-values are much lower than 0.05, with that of BG being lower only by one order of magnitude. PU remains within null hypothesis with its PCC = −0.11 and its *p*-value = 0.59.

**Figure 9:**
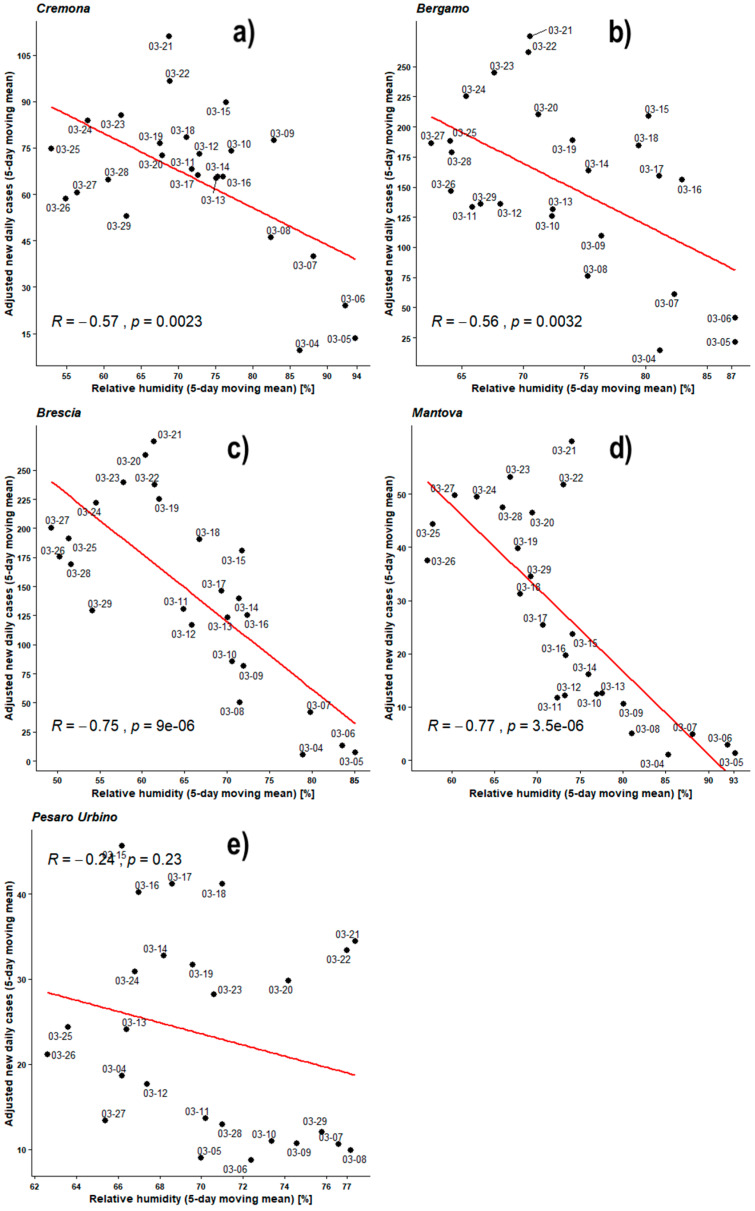
Scatter plot and linear regression for the 5-day moving means of adjusted new daily cases of COVID-19 against relative humidity for five Italian provinces: (**a**) Cremona, (**b**) Bergamo, (**c**) Brescia, (**d**) Mantova, (**e**) Pesaro Urbino. Labels report, in MM-DD format, the last day of the moving means. Pearson correlation coefficient is reported as ‘R’ while probability value is reported as ‘*p*’.

Figure 9 shows Pearson’s analyses regarding the 5-day moving means of daily new infected individuals, adjusted against the number of individuals checked in the respective region, and outdoor daily average humidity. All observed PCCs are negative in the relative humidity range 50–95%. The PCCs of BG, BS, CR, and MN are between −0.56 and −0.77 while all the *p*-values remain lower than 0.05, with those of BG and CR being lower only by one order of magnitude. PU remains within null hypothesis with its PCC = −0.24 and its *p*-value = 0.23.

For both previous Pearson’s analyses (humidity against new daily cases, and humidity against adjusted new daily cases) linear correlation trends are always negative in the relative humidity range 50–95%. During the analyses against the outdoor temperatures, normalizing against the number of checks led to a smoothing and a general improvement of results. Here the same analyses do not show a homogeneous improvement of the correlations, and for CR, BS, and MN, there is a drop in the correlation coefficient from −0.68, −0.81, and −0.82 respectively to −0.57, −0.75, and −0.77. BG only shows a slight increase in PCC going from −0.54 to −0.56. PU always remains uncorrelated.

**Figure 10:**
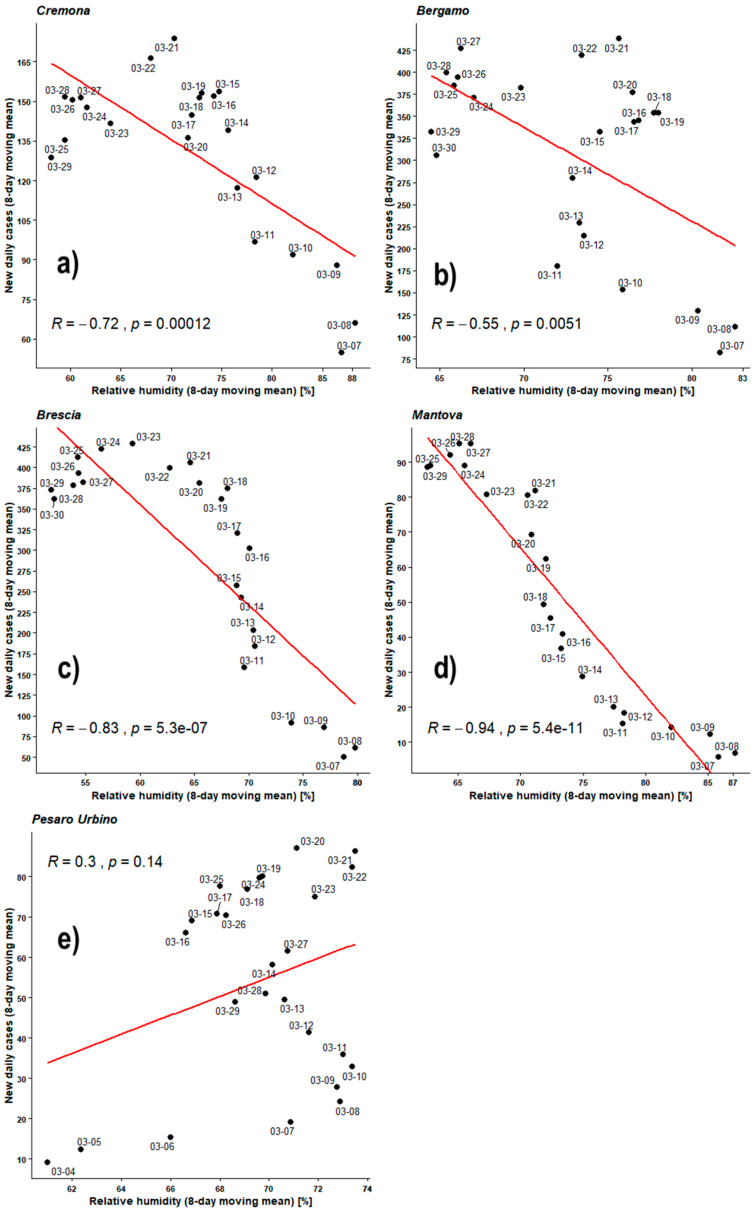
Scatter plot and linear regression for the 8-day moving means of new daily cases of COVID-19 against relative humidity for five Italian provinces: (**a**) Cremona, (**b**) Bergamo, (**c**) Brescia, (**d**) Mantova, (**e**) Pesaro Urbino. Labels report, in MM-DD format, the last day of the moving means. Pearson correlation coefficient is reported as ‘R’ while probability value is reported as ‘*p*’.

Figure 10 shows the Pearson’s analyses performed over daily average relative humidity time series and recently infected individual time series both averaged by the 8-day moving mean. All observed PCC are negative except for the PU one, which turns positive probably due to the concentration of almost all the experimental points within an even narrower humidity range. PCC values of CR, BS, and BG are in the range −0.55 to −0.83. All *p*-values are much lower than the 0.05 except for the BG one, which is equal to 0.0051. This time MN shows a very strong correlation, the PCC being −0.94 with an impressing *p*-value of 10^−9^ order of magnitude.

As already outlined, PU shows a positive correlation but its *p*-value shows a very weak statistics.

**Figure 11:**
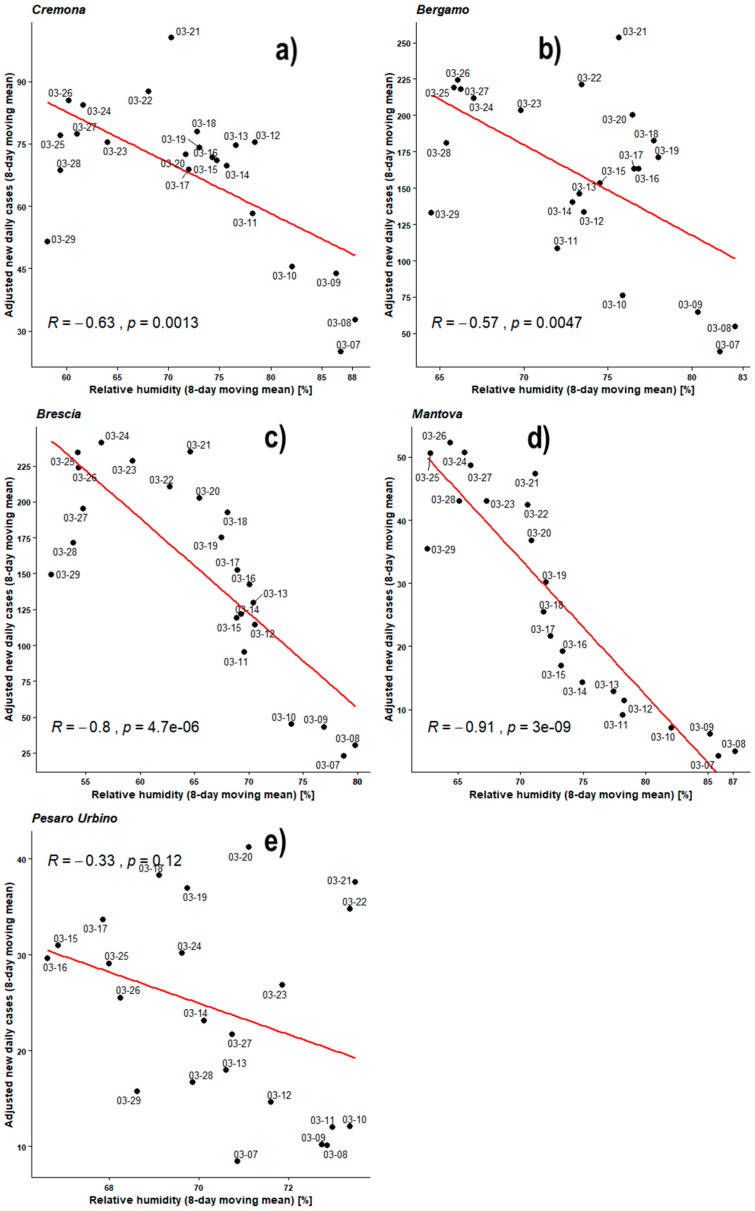
Scatter plot and linear regression for the 8-day moving means of adjusted new daily cases against relative humidity for five Italian provinces: (**a**) Cremona, (**b**) Bergamo, (**c**) Brescia, (**d**) Mantova, (**e**) Pesaro Urbino. Labels report, in MM-DD format, the last day of the moving means. Pearson correlation coefficient is reported as ‘R’ while probability value is reported as ‘*p*’.

Figure 11 shows Pearson’s analyses regarding the 8-day moving means of daily new infected individuals, normalized against the number of individuals checked, and outdoor daily average humidity. This time, all observed PCC are negative, including PU’s one. The PCCs of BG, BS, CR, and MN are between −0.57 and −0.91 while all the *p*-values remain lower than 0.05 with those of BG and CR being lower by one order of magnitude only. PU remains within null hypothesis with its PCC = −0.33 and its *p*-value still unacceptable.

### 3.3. Resume of the Correlations between the Moving Mean of New Daily Cases and the Meteorological Parameters

Table 3 shows the correlations between the moving mean of new daily cases and the meteorological parameters. In the time period considered, the five provinces had a mean daily temperature of 9.8 °C, and mean relative humidity of 70.9%. For all the five provinces, positive correlations were observed between the moving means of new daily cases and the moving means of outdoor temperatures in the range 5–14 °C. However, most correlations were not statistically significant for MN, and PU.

Multiple regression analysis was also performed with case rate as the dependent variable and temperature plus relative humidity as independent variables. Here we only summarize results in Table 4. The table shows multiple R, intercept stat t, temperature stat t, relative humidity stat t, and the related *p*-values for the “unadjusted” infection-rate time series. Results are in good agreement with those of Pearson’s analyses. Statistical correlations are evident in BG, BS, CR, and MN while PU still shows values very near to null hypothesis condition. MN now shows multiple R much closer to those of BG, BS, and CR but the *p*-values related to temperature are rather high, while those related to relative humidity are very good. This seems to confirm that MN statistics are close to those of other Lombardy provinces but not equivalent. Results for “adjusted” infection-rate time series are very similar. Regarding F-test results, F-stat values are in the range 20 to 40 for BG, CR, and MN while peak up to 260 for BS but fall down to 1 for PU. Accordingly, the related significances are in the range 10^-10^–10^-6^ for BG, CR and MN, in the range 10^−15^–10^−11^ for BS and in the range 10^−2^–10^−1^ for PU.

## 4. Discussion

The positive correlations calculated between COVID-19 incidence and the outdoor temperatures were in line with previous studies about COVID-19 pandemic in New York (United States) [38], Jakarta (Indonesia) [39], and various cities and regions around the world [21]. The range of outdoor temperatures (5–14 °C), that was observed in the present study for the five provinces of Italy, is in the range of temperatures (3–17 °C) that was reported by Bukhari and Jameel [40] for the countries with more than 90% of COVID-19 new cases across the world.

Bashir et al. [38] have reported significant positive correlations between COVID-19 total daily cases and mean daily temperature in the range 1.8–15.2 °C. Furthermore, Tosepu et al. [39] have pointed out similar findings, with a significant positive correlation between the new daily cases and mean daily temperatures in the range 26.1–28.6 °C.

In various cities and region around the world, new daily positive cases significantly increased with increasing mean daily temperatures of up to 8.7 °C [21].

Our results are in contrast with some previous studies [15,17,41,42] regarding a negative linear relationship between outdoor temperature and COVID-19 incidence. Yet, comparisons with previous studies should take into account the different meteorological conditions, since the occurrence of SARS-CoV-2 outbreak has been observed for a wide range of temperatures around the globe. For example, Rahman et al. [20] have observed negative correlations between outdoor temperature and SARS-CoV-2 infectivity statistically significant for provinces with a median temperature of −2.9 °C. Moreover, increases in daily mean temperature between 3 and 21 °C did not affect the containment of the COVID-19 outbreak in Wuhan (China) [43].

Negative correlations were observed between the moving means of new daily cases and the moving means of outdoor relative humidity in the range 50–95% for more than 90% of the infected. The correlation between the PU new daily cases, thus for about 6.8% of the total number of infected, and relative humidity were not statistically significant. Negative relationships between relative humidity and COVID-19 incidence have been reported also in previous studies about several Chinese cities [17] and countries around the globe [15].

According to Chen et al. [13], the trend of new daily positive is better reproduced by the models that combine several meteorological parameters (e.g., air temperature, wind speed, visibility, and relative humidity) rather than correlations with a single meteorological variable.

The PCC, the related *p*-values and the results of multiple regression analyses associated with the three central provinces of Lombardy (BG, BS, and CR) and, in most cases those of MN, show strong correlations between the selected meteorological parameters and the trend of COVID-19 new infections. This strong correlation is positive for temperature and this means that infections were more likely to occur at higher temperatures in the range about 5–14 °C. Such temperature range refers to the 5-day moving mean and the 8-day moving mean, so the actual range is slightly wider. The same strong correlation is negative for relative humidity and this means that infections were more likely to occur at low levels of humidity in the range about 50–95%. Again, such range of humidity refers to the 5-day moving mean and the 8-day moving mean, so the actual range is slightly wider. As already stressed, correlation does not imply causation, so it remains difficult to find a possible explanation for such statistical evidence. A simple possible explanation could be the need of a close contact for the infection to occur combined with the tendency of most people, especially elderly people, to gather more frequently when the weather is fair. Thus, we can expect more narrow contacts and infections in warmer dry days. As an example, Italian news reported about records of victims within bocce-ball players, who are mostly elderly people often playing outdoor.

The PU province did not show such strong statistical evidence. Correlations between temperatures and infections exist and are still positive, but the degree of confidence is much lower. The correlation between humidity and infections is simply null for PU. However, such statistics might have been weakened by the reduced temperature range and the very narrow humidity range. In fact, after averaging, almost all relative humidity values were in the range 65–75%.

## 5. Limitations

The first limitation of this study is represented by the reduced number of provinces considered. All the provinces showing significant statistics belong to the central-upper Po Valley. A comparison with other regions showing similar trends of infections and comparable meteorological conditions is crucial for validating our results.

The second limitation of this study is represented by the short time span considered. As a matter of fact, temperatures tend to increase steadily in March and so the trend of infections also increased during the entire month. However, at the end of the month a sharp decrease in temperature and humidity occurred due to a penetration of Artic cold and dry air masses. This decrease is visible in Figure 2 and Figure 3. Since we had already developed statistical analyses, we were concerned about this point. However, once the new data were introduced, to our surprise, we did not find a significant decrease in PCCs and *p*-values.

Another possible source of errors derives from the assessment of COVID-19 victims. We know that many victims were not included within statistics since they did not have the time or the possibility of being checked for SARS-CoV-2. Some researchers postulate an overall much higher number of victims. Furthermore, within the three most infected areas, records of infections happened within residences for the elderly and chronically ill, and this might have altered statistics.

More in general, epidemiological data could be affected by errors both at the regional and national level as pointed out in previous works [34,35]. Additionally, the so-called “adjusted” number of infections was extrapolated somehow combining the number of infections within provinces with the number of tests performed in the same region. This was inevitable since we do not have the latter data collected at province level. Such data will probably never be published as there are problems in identifying precisely the areas of samplings given that, in most cases, patients were asked to reach collection sites and the samples were sent to laboratories often far from there.

## 6. Conclusions

The present study investigated the statistical correlation between meteorological parameters, namely, daily mean temperature and mean relative humidity, and the incidence of COVID-19 for a 30-day time period (from 29th of February 2020 to 29th of March 2020) in Italy. Five provinces were selected according to the number of infected individuals in the early stages of the COVID-19 epidemic. In the time period considered, these provinces reported 23,184 new cases of COVID-19. In particular, Bergamo and Brescia showed some of the highest trends of infections, while nearby Cremona and Mantova, showed lower trends and were selected to check statistical evidence under different conditions. Finally, Pesaro–Urbino province was included for further investigation as it was comparably affected by the epidemic despite being the area far from the Po Valley. Furthermore, the selected provinces represent three different climatic areas of Italy. In the time period considered, these provinces had a mean daily temperature of 9.8 °C and mean relative humidity of 70.9%.

Moving means were calculated at 5 days and 8 days, since the statistics of the new daily positive cases may be affected by the length of the incubation period and by uncertainties in the epidemiological data. For a second set of experiments, the number of new daily positive cases was adjusted considering possible variations in the daily number of specimens collected, biasing per each province the number of new daily positive cases for the ratio between the new daily positive cases and the total number of tests performed in the respective region.

For each province, adjusted and unadjusted new daily cases were plotted versus the meteorological parameters, and linear regressions were determined. For the five provinces of Italy, strong positive correlations were observed between the moving means of new daily cases and the moving means of daily temperatures. However, most correlations were not statistically significant for MN and PU. Strong negative correlations were observed between the moving means of new daily cases and moving means of relative humidity for more than 90% of the infected. The correlation between the PU new daily cases and relative humidity were not statistically significant. This seems to indicate a general positive correlation between COVID-19 rates of contagion and the outdoor temperatures in the range 5–14 °C, and a general negative correlation between COVID-19 rate of contagion and outdoor humidity in the range 50–95%.

This study is limited to the analysis of meteorological parameters for a 30-day time period. It is likely that several other factors concur in determining the incidence of COVID-19. Therefore, it is not possible to consider outdoor temperature and relative humidity as driving factors of the COVID-19 incidence. A robust model linking all the driving factors to the experimental evidence would be useful to support the statistical analyses. In this context, we did not consider any forecasts regarding the propagation of the SARS-CoV-2 to be suitable and/or realistic.

## Figures and Tables

**Table 1:** Statistics of Coronavirus Disease-2019 (COVID-19) contagion in the selected provinces from 29 February 2020–29 March 2020.

Region	Province	Total Infected Per Province	Total Infected Per Region	Total Tests Per Region
Lombardy	Bergamo	8424	40,476	102,563
	Brescia	8000		
	Cremona	3639		
	Mantova	1550		
Marche	Pesaro Urbino	1571	3154	10,384

**Table 2:** Location of the monitoring stations in northwest and central Italy.

Region	Province	Latitude	Longitude	Elevation above Sea Level [m]
Lombardy	Cremona	45°08′35′′	10°02′49′′	40
	Bergamo	45°42′51′′	9°41′45′′	284
	Brescia	45°30′52′′	10°13′02′′	122
	Mantova	45°09′45′′	10°49′03′′	34
Marche	Pesaro Urbino	43°54′41.767′′	12°53′2.965′′	40

**Table 3:** The Pearson correlation coefficients (‘R’) and the *p*-values (‘*p*’) obtained though Pearson’s analyses over new daily cases of COVID-19 and meteorological parameters for five provinces of Italy in the time period from the 29th of February 2020 to the 29th of March 2020.

Pearson’s Analysis	Cremona	Bergamo	Brescia	Mantova	Pesaro Urbino
Temperature versus new daily cases (5-day moving mean)	R = 0.75	R = 0.65	R = 0.7	R = 0.21 *	R = 0.26 *
	*p* = 7.2 × 10^−6^	*p* = 2.7 × 10^−4^	*p* = 5.6 × 10^−5^	** *p* ** = 0.3 *	** *p* ** = 0.2 *
Temperature versus adjusted new daily cases (5-day moving mean)	R = 0.74	R = 0.71	R = 0.75	R = 0.29 *	R = 0.52
	*p* = 1.6 × 10^−5^	*p* = 4.7 × 10^−5^	*p* = 1.1 × 10^−5^	** *p* ** = 0.15 *	*p* = 6.3 × 10^−3^
Temperature versus new daily cases (8-day moving mean)	R = 0.83	R = 0.73	R = 0.79	R = 0.28 *	R = 0.065 *
	*p* = 8.1 × 10^−7^	*p* = 4.5 × 10^−5^	*p* = 4.3 × 10^−6^	** *p* ** = 0.19 *	** *p* ** = 0.75 *
Temperature versus adjusted new daily cases (8-day moving mean)	R = 0.81	R = 0.77	R = 0.83	R = 0.33 *	R = 0.45
	*p* = 2.8 × 10^−6^	*p* = 2 × 10^−5^	*p* = 1.2 × 10^−6^	** *p* ** = 0.13 *	*p* = 0.03
Relative humidity versus new daily cases (5-day moving mean)	R = −0.68	R = −0.54	R = −0.81	R = −0.82	**R =** **−** 0.11 *
	*p* = 1.1 × 10^−4^	*p* = 4 × 10^−3^	*p* = 3.5 × 10^−7^	*p* = 1.2 × 10^−7^	** *p* ** = 0.59 *
Relative humidity versus adjusted new daily cases (5-day moving mean)	R = −0.57	R = −0.56	R = −0.75	R = −0.77	**R =** **−** 0.24 *
	*p* = 2.3 × 10^−3^	*p* = 3.2 × 10^−3^	*p* = 9 × 10^−6^	*p* = 3.5 × 10^−6^	** *p* ** = 0.23 *
Relative humidity versus new daily cases (8-day moving mean)	R = −0.72	R = −0.55	R = −0.83	R = −0.94	R = 0.3 *
	*p* = 1.2 × 10^−4^	*p* = 5.1 × 10^−3^	*p* = 5.3 × 10^−7^	*p* = 5.4 × 10^−11^	** *p* ** = 0.14 *
Relative humidity versus adjusted new daily cases (8-day moving mean)	R = −0.63	R = −0.57	R = −0.8	R = −0.91	**R =** **−** 0.33 *
	*p* = 1.3 × 10^−3^	*p* = 4.7 × 10^−3^	*p* = 4.7 × 10^−6^	*p* = 3 × 10^−9^	** *p* ** = 0.12 *

* Values indicating no significant Pearson correlation are shown in bold red while the only trend contrary to the common statistics is shown in bold violet.

**Table 4:** Selected indices deriving from multiple regression analysis applied over new daily cases of COVID-19 vs. meteorological parameters for five provinces of Italy in the time period from the 29th of February 2020 to the 29th of March 2020.

Multiple Regression Analysis		Cremona	Bergamo	Brescia	Mantova	Pesaro Urbino
Temperature and relative humidity vs. new daily cases (5-day moving mean)	M.R	0.812	0.812	0.948	0.834	0.272
	I.s.t	4.286	3.403	5.932	6.265	0.488
	T.s.t	6.358	5.003	7.462	1.136	1.235
	R.H.s.t	−5.320	−4.028	−9.755	−7.184	−0.355
	I.p	2.55 × 10^−4^	2.44 × 10^−3^	4.78 × 10^−6^	1.78 × 10^−6^	6.30 × 10^−1^
	T.p	1.42 × 10^−6^	4.62 × 10^−5^	1.39 × 10^−7^	2.67 × 10^−1^	2.29 × 10^−1^
	R.H.p	1.85 × 10^−5^	5.25 × 10^−4^	1.22 × 10^−9^	2.00 × 10^−7^	7.26 × 10^−1^
Temperature and relative humidity vs. new daily cases (8-day moving mean)	M.R	0.910	0.891	0.980	0.938	0.349
	I.s.t	2.353	3.419	5.872	9.402	−1.376
	T.s.t	6.053	7.061	12.150	−0.716	0.915
	R.H.s.t	−3.937	−5.109	−13.490	−11.508	1.756
	I.p	2.89 × 10^−2^	2.58 × 10^−3^	7.90 × 10^−6^	8.84 × 10^−9^	1.82 × 10^−1^
	T.p	6.45 × 10^−6^	5.73 × 10^−7^	5.79 × 10^−11^	4.82 × 10^−1^	3.70 × 10^−1^
	R.H.p	8.15 × 10^−4^	4.64 × 10^−5^	8.17 × 10^−12^	2.84 × 10^−10^	9.25 × 10^−2^

Indices are: multiple R (M.R); intercept stat t (I.s.t), and *p*-value (I.p); temperature stat t (T.s.t), and *p*-value (T.p); relative humidity stat t (R.H.s.t), and *p*-value (R.H.p).
